# Fatal Duodenal Ulcer Hemorrhage in a Post-Operative Patient of Proximal Femur Fracture Undergoing Carbon Ion Radiotherapy for Locally Advanced Pancreatic Carcinoma: A Case Report

**DOI:** 10.7759/cureus.86472

**Published:** 2025-06-21

**Authors:** Hiroki Iida, Mikako Suzuki, Hiroya Mizokami, Ryu Kondo, Hideki Takagi

**Affiliations:** 1 Orthopaedic Surgery, Nagoya Central Hospital, Nagoya, JPN; 2 Orthopaedic Surgery, Nagoya Nishi Hospital, Nagoya, JPN

**Keywords:** duodenal ulcer disease, heavy ion radiotherapy, outcomes of hip fracture, proximal femur fracture, unresectable pancreatic cancer

## Abstract

The incidence and mortality rates of pancreatic cancer have been increasing. Due to the high likelihood of metastasis and vascular invasion, most pancreatic cancer cases are inoperable at the time of diagnosis. Carbon-ion radiotherapy (CIRT) has recently been introduced as a treatment option for locally advanced pancreatic cancer (LAPC). However, potential complications, including peptic ulcer disease (PUD), have been noted. This report presents a case of death due to PUD following a proximal femur fracture, with a history of CIRT for unresectable LAPC.

A 59-year-old female undergoing CIRT for unresectable LAPC sustained a proximal femur fracture due to a fall. She underwent open reduction and intramedullary nailing. The surgery was performed successfully, but on the third postoperative day, she developed massive gastrointestinal bleeding due to a duodenal ulcer. Despite initial stabilization, a recurrence of the bleeding occurred on the fifth postoperative day, and she passed away.

This case highlights the potential association between CIRT and PUD, emphasizing the need for careful perioperative gastrointestinal management in patients with a history of CIRT.

## Introduction

The age-standardized mortality rate for all cancers has continued to decrease; however, both the incidence and mortality rates of pancreatic cancer have been increasing [1]. Approximately 80% of pancreatic cancer cases are inoperable at the time of diagnosis due to the high likelihood of metastasis to peripancreatic lymph nodes and invasion of major blood vessels [2]. In recent years, radiotherapy has played an increasingly important role in the treatment of pancreatic cancer [3]. High-energy photons are commonly used in radiation therapy for malignant tumors. They deliver their maximum dose to the body surface, attenuating the dose as they penetrate deeper into the lesion.

In contrast, particle beams, such as protons and carbon ions, exhibit a Bragg peak, a phenomenon in which the energy deposition of charged particles is minimal along their path and rises sharply at a specific depth, just before the particles come to rest. This property enables better sparing of surrounding healthy tissues compared to conventional X-rays. 

Furthermore, carbon ions have a higher relative biological effectiveness (RBE), which refers to the ratio of the dose of reference radiation (usually X-rays) to the dose of the radiation required to achieve the same biological effect. Carbon ions have an RBE approximately two to three times that of X-rays [4]. This means that a lower radiation dose can achieve the same, or even greater, tumor cell-killing effects, making carbon-ion therapy particularly effective against radiation-resistant tumors.

Carbon-ion radiotherapy (CIRT) is a type of particle therapy that utilizes carbon ions to deliver highly localized and potent radiation doses to tumors. Due to its superior dose distribution and enhanced biological effect, CIRT has been increasingly used for treating tumors that are difficult to resect surgically or are resistant to conventional radiotherapy. In Japan, CIRT for unresectable locally advanced pancreatic cancer (LAPC) was approved for insurance coverage in April 2022 and is now included in pancreatic cancer treatment guidelines. However, several studies have reported an association between CIRT for pancreatic cancer and peptic ulcer disease (PUD).

Here, we report a case of death due to PUD following a proximal femur fracture, potentially associated with a history of CIRT for unresectable LAPC.

## Case presentation

A 59-year-old female patient undergoing CIRT for an unresectable LAPC over the past two years at another hospital has a medical history that includes chronic kidney disease and type 2 diabetes. CIRT was administered solely to the pancreatic tumor with no extension to adjacent organs. The prescribed dose of CIRT was 55.2 Gy (RBE). She sustained a proximal femur fracture due to a fall and was initially hospitalized at a nearby general hospital. The next day, the patient was transferred to our hospital. Upon admission, a blood test revealed a low hemoglobin level of 7.2 g/dL, and the patient received four units of red blood cell transfusion. No gastrointestinal symptoms were noted preoperatively; therefore, prophylactic PPI use was not initiated. Open reduction and intramedullary nailing were performed under general anesthesia on the following day. Perioperative thromboprophylaxis included the use of compression stockings and intermittent pneumatic compression devices. Pharmacologic prophylaxis was not administered due to the patient’s renal impairment.

**Figure 1:**
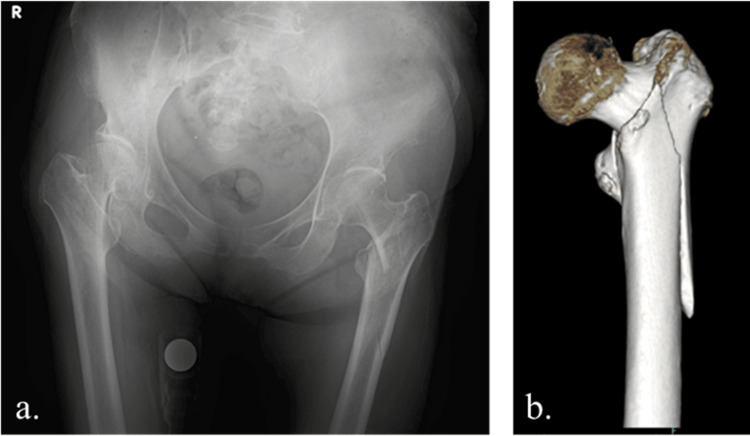
Preoperative X-ray and three-dimensional computed tomography image. a. Preoperative X-ray image. b. Preoperative three-dimensional computed tomography image. A proximal femur fracture was observed in the left hip.

**Figure 2:**
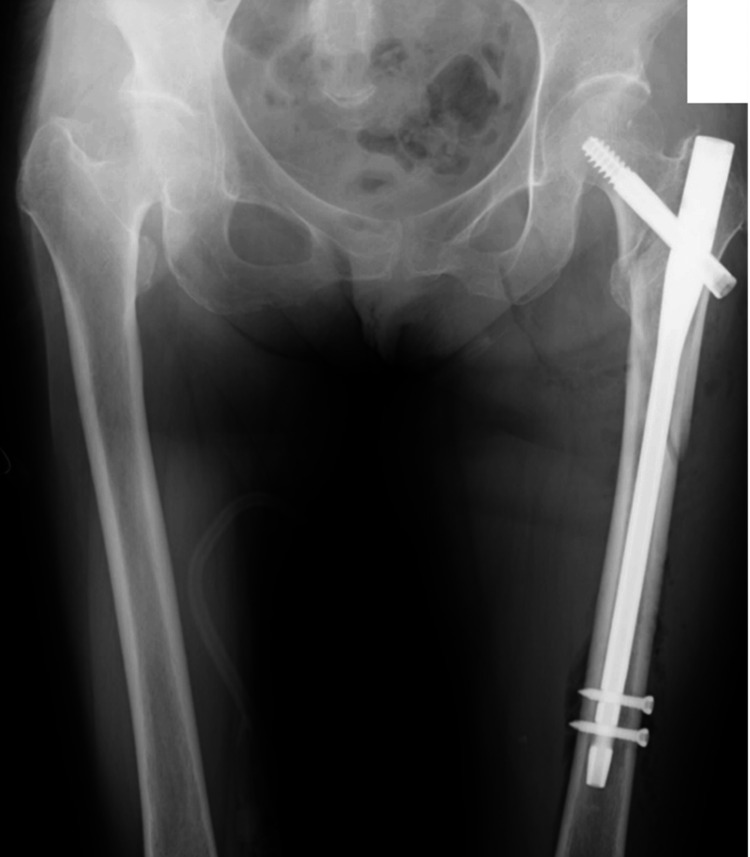
Postoperative X-ray The surgical procedure involved an IPT-EF nail (125°×260mmφ10mm; HOMS Engineering Inc., Nagano, Japan), and the NESPLON Cable System (Alfresa Pharma Corporation, Osaka, Japan) was used for subtrochanteric fixation.

Figure 1 shows preoperative X-ray and three-dimensional computed tomography images. The findings did not suggest a metastatic bone tumor. The operation was performed using a traction table in the supine position. The operating time was 89 minutes, with an estimated blood loss of 110 ml. The surgical procedure involved an IPT-EF nail (125°×260mmφ10mm; HOMS Engineering Inc., Nagano, Japan), and the NESPLON Cable System (Alfresa Pharma Corporation, Osaka, Japan) was used for sub-trochanteric fixation (Figure 2). The day after surgery, the patient was allowed to load the operated limb and began gait training.

**Figure 3:**
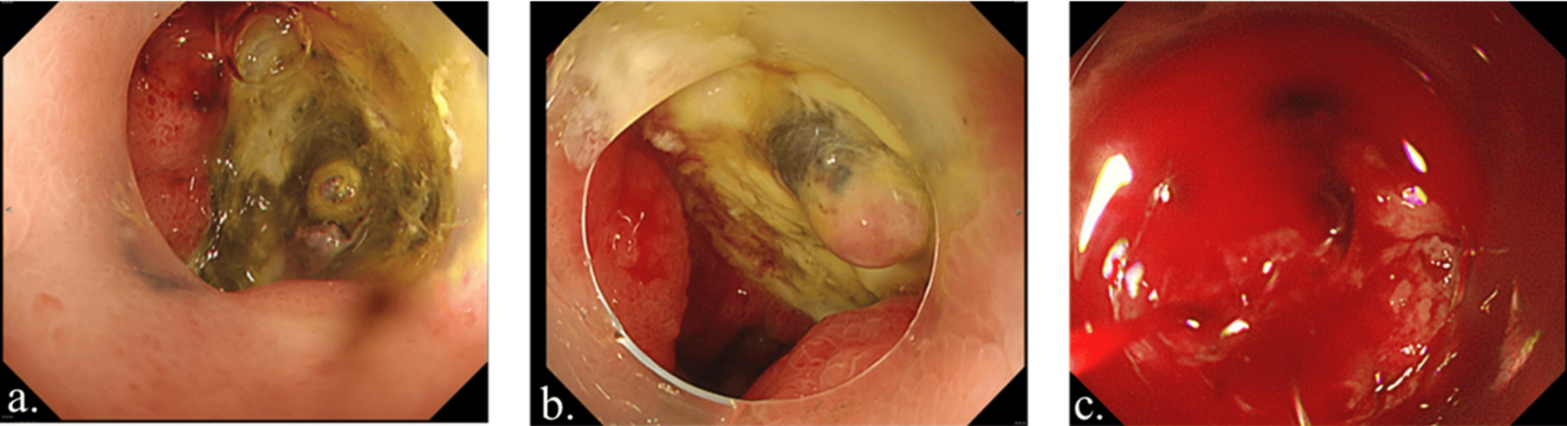
Upper gastrointestinal endoscopy findings are shown. a. On the third postoperative day, an upper gastrointestinal endoscopy was performed, and the patient was diagnosed with a duodenal ulcer. b. On the second upper gastrointestinal endoscopy performed on the fifth postoperative day, there was no recurrent bleeding, and a white coating was observed at the base of the ulcer, which was in the healing phase. c. On the sixth postoperative day, emergency upper gastrointestinal endoscopy revealed active spurting hemorrhage from the base of the duodenal ulcer.

On the third postoperative day, abdominal pain and massive melena were present. Blood tests revealed hemoglobin of 3.8 g/dL, suspected PUD. An emergency upper gastrointestinal endoscopy was performed and diagnosed a duodenal ulcer (Figure 3a). However, since no active bleeding was observed, the hemostatic intervention was not performed due to the risk of rebleeding. The patient received six units of red blood cell transfusion, and proton pump inhibitor (PPI) administration was initiated with fasting instructions. The prothrombin time-international normalized ratio (PT-INR) was 1.18, and the activated partial thromboplastin time (APTT) was 26.2 seconds, indicating that the patient was not in a hemorrhagic state.

On the second upper gastrointestinal endoscopy performed on the fifth postoperative day, there was no recurrent bleeding, and a white coating was observed at the base of the ulcer, which was in the healing phase (Figure 3b). However, in the early hours of the next day, the patient experienced a recurrence of massive duodenal ulcer bleeding, leading to shock. An emergency upper gastrointestinal endoscopy revealed an active spurting hemorrhage from the base of the duodenal ulcer (Figure 3c). Hemostatic intervention was attempted, but the procedure proved challenging due to the severity of the bleeding. During the endoscopy, the patient’s condition progressively worsened, with a significant drop in blood pressure culminating in pulseless electrical activity. Despite resuscitation efforts, including cardiopulmonary resuscitation and massive blood transfusion, the patient could not recover and passed away on the same day.

## Discussion

Proximal femur fractures significantly reduce life expectancy and substantially interfere with activities of daily living [5,6]. Patients with pancreatic cancer who sustain a proximal femur fracture are expected to have an even worse prognosis.

The median survival time for unresectable pancreatic cancer is 8.2 months; however, in this case, the patient survived for more than two years with CIRT. Cancer survivors are at a higher risk of proximal femur fractures [7,8] and have a poor prognosis [9]. In our case, the patient developed a postoperative duodenal ulcer, which ultimately led to death.

The stomach and duodenum are in close proximity and, therefore, are susceptible to the adverse effects of radiation therapy for pancreatic cancer. Several studies have reported an association between CIRT for pancreatic cancer and gastrointestinal ulcers and bleeding. The Common Terminology Criteria for Adverse Events (CTCAE) Version 5.0, developed by the National Cancer Institute, is an internationally accepted standard for the classification and grading of adverse events associated with cancer therapy. The severity of each adverse event is categorized as follows:

Grade 1 (Mild): Asymptomatic or mild symptoms; clinical or diagnostic observations only; intervention not indicated. Grade 2 (Moderate): Moderate symptoms; minimal, local, or noninvasive intervention indicated; limiting age-appropriate instrumental activities of daily living (ADL). Grade 3 (Severe): Severe or medically significant but not immediately life-threatening; hospitalization or prolongation of hospitalization indicated; disabling; limiting self-care ADL. Grade 4 (Life-threatening): Life-threatening consequences; urgent intervention indicated. Grade 5 (Death): Death related to the adverse event.

The incidence of Grade 1 and 2 gastric ulcers following CIRT for pancreatic cancer is reported to be 21% [10], while the incidence of gastrointestinal bleeding and acute ulcers of Grade 3 or higher ranges from 1.3% to 4.7% [11,12]. 

Although the patient did not use nonsteroidal anti-inflammatory drugs (NSAIDs) due to a medical history of chronic kidney disease, NSAIDs are a well-known risk factor for PUD. The odds ratio for developing gastrointestinal ulcers due to NSAID use is 19.4 [13]. Additionally, the relative risk of upper gastrointestinal bleeding or perforation in the NSAID group was 4.5 times that of the non-NSAID group [14]. Moreover, the incidence of PUD associated with proximal femur fractures is reported to be 1.3% to 2% [15,16]. Based on these findings, cancer survivors with proximal femur fracture may have a relatively higher risk of PUD as a postoperative complication.

PPIs are commonly used in the treatment of PUD and prevent rebleeding [17]. In this case, we initiated PPI therapy immediately after the diagnosis of a duodenal ulcer; however, rebleeding occurred. Inadomi J et al. reported that 82% of patients who had a recurrence of bleeding within eight days after endoscopy required surgery [18]. Therefore, surgical intervention might have been warranted at the time of the second bleeding episode. However, the mortality rate of patients who underwent surgery for PUD is 20.0% [19]. Moreover, surgical intervention after radiation therapy is challenging due to fibrosis of adjacent organs. Consequently, even if surgical intervention was performed, improving this case's outcome might have been difficult. Since the family did not consent to a pathological autopsy, we were unable to confirm the presence of tumor invasion or fibrosis around the ulcer. Fujishiro T et al. reported a case of duodenal stenosis after CIRT for LAPC [20]. Nevertheless, the possibility that CIRT leads to PUD in this case cannot be excluded. Further research is needed on the prescribed radiation dose and the late effects on each target organ in CIRT.

Although a causal relationship between the aftereffects of proximal femur fracture surgery and the aftereffects of heavy ion beam therapy remains unclear, it is also important to note that the patient’s comorbidities may have contributed significantly to the development and severity of the ulcer. This case highlights the importance of vigilant gastrointestinal risk assessment in postoperative care for patients with a history of abdominal radiotherapy, even in the absence of NSAID use.

## Conclusions

We reported a case of fatal duodenal ulcer hemorrhage following a proximal femur fracture in a patient who had undergone CIRT for unresectable LAPC. This case suggests that CIRT may contribute to an increased risk of PUD, potentially leading to fatal complications. Considering the potential association between CIRT and PUD, clinicians should be aware of this risk, especially in patients with additional factors such as trauma or fractures. Further research is needed to better understand the mechanisms underlying this association and to establish optimal management strategies for high-risk patients.
